# Acute Whole Body Vibration Decreases the Glucose Levels in Elderly Diabetic Women

**DOI:** 10.1155/2018/3820615

**Published:** 2018-06-05

**Authors:** Maíra Florentino Pessoa, Helga C. Muniz de Souza, Alanna P. Vasconcelos da Silva, Rafaela dos Santos Clemente, Daniella Cunha Brandão, Armèle Dornelas de Andrade

**Affiliations:** Department of Physical Therapy, Universidade Federal de Pernambuco, 50.740-560 Recife, PE, Brazil

## Abstract

Type II diabetes (TIIDM) is characterized by high levels of blood glucose followed by excessive insulin release so that the target cells become less sensitive, developing insulin resistance and maintaining hyperglycemic levels. Physical activity is the strongest element to prevent and to manage the TIIDM, and the majority of patients do not remain in regularly active levels, because the premature fatigue in these patients decreases the adherence to the training. Contrastingly, the whole body vibration (WBV) training may improve the glucose metabolism in diabetic patients, reducing the peripheral blood sugar, decreasing the physical discomfort and perceived exertion. Therefore, the purpose of the study was to determine the effect of an acute WBV session as therapy to promote fasting decreases in insulin levels in peripheral blood in TIIDM when compared to healthy elderly. For this, fifteen healthy elderly women and fourteen diabetic elderly women, all sedentary, were allocated in diabetic or control groups, and we made an acute whole body session composed of 10 bouts lasting 2 minutes each one, separated by a 30-second rest period. The WBV was executed in a triaxial platform MY3 Power Plate® at 35 hertz and has been chosen a peak-to-peak displacement of 4 millimeters. After the protocol, both groups decreased the glycemic levels and increased lactate production in relation to the basal levels and when compared diabetic and control, where the most important results have been shown in diabetic women. This study revealed that WBV training in TIIDM has had significant beneficial effects on the control of glucose levels, still in an acute session. So that, the complete training probably will show better results about glycemic control and this finding could be especially important when prescribing exercise for elderly who are unable or unwilling to use traditional loads or who show poor exercise compliance.

## 1. Introduction

According to International Diabetes Center, the type II diabetes (TIIDM) is the most common diabetes type, whose prevalence is growing around 300% from 1980 to 2010 [1]. This disease is determined by an interplay between genetic and metabolic factors combined, being the overweight, the physical inactivity, and the aging, stronger risks factors [2] with the majority of deaths occurring between 60 and 69 years [1].

The TIIDM is characterized by high levels of blood glucose followed by excessive insulin release so that the target cells become less sensitive, developing insulin resistance and maintaining hyperglycemic levels [3]. During the insulin stimulation, an insulin-dependent transporter, the glucose transporter type 4 (GLUT4), abundant in skeletal muscle cells and adipocytes, is translocated across the cell membrane, increasing the glucose transport. The insulin resistance occurs when there are problems in transduction of the insulin-induced signal, generating failings in translocation pathway of GLUT4 that remain in vesicles into the cytoplasm [4].

However, according to American Diabetes Association (ADA), modest changes in diet and mainly in physical activity levels are precise and efficient for reducing the TIIDM incidence around 50% in people with impaired glucose regulation [5]. The muscle contraction is the first method to improve the GLUT4 translocation [6] and although physical activity is the strongest element to prevent and to manage the TIIDM, the majority of patients with this chronic disease do not remain in regularly active levels, because they are easily fatigued even during the mild exercises [7]. Moreover, strenuous physical activity in sedentary individuals promotes physical discomfort, decreasing the exercise adherence [8].

A joint position between the American College of Sports Medicine and the ADA published a statement about exercise effects on glucose levels in TIIDM. This study shows with C level of evidence that acute resistance exercise results in lower fasting blood glucose levels for at least 24 hours after exercise, but only in moderate to vigorous exercises. In relation to acute aerobic exercises, the results show that their action could be different according to intensity, with C level of evidence. Moderate intensities reduce the glucose levels between 24 and 72 hours and intense aerobic exercises can even increase the glucose production, by raises in plasma catecholamine levels, with hyperglycemia persisting for up to 1-2 hours. Based on these results, the joint statement recommends the combination between aerobic and resistance exercises, with B level of evidence [9].

However, an exercise intervention for elderly with TIIDM requires being as efficient and fast as possible, permitting the benefits of moderate intensity without allowing the fatigue installation or joint pain. For these patients, the whole body vibration (WBV) appears as a novel and useful regime training, stimulating the muscle contraction through the muscle spindle activation and stretch-reflex activation [10]. The vibrating platform may be a promising modality because it does not cause joint injuries [11] and associates resistance and aerobic training, achieving contractions through tonic vibration reflexes in the whole body. This training modality could be considered a moderate exercise for subjects with low physical activity levels, generating the same results as resistance training about the glucose uptake in muscle, resulting in improved insulin sensitivity for diabetic patients [12].

In addition, the time related to WBV session is notably less than traditional resistance exercises [13]; besides presenting lower levels of perceived exertion [14] and physical discomfort [15], the WBV could show a potential for feasible reduction in acute pharmacologic usage for this population.

In the light of the above-mentioned, it was hypothesized that an acute WBV session could improve the glucose metabolism in diabetic patients by reducing the peripheral blood sugar. With this in mind, the purpose of the present study was to determine the effect of an acute WBV session as additional therapy to promote fasting decreases in insulin levels in peripheral blood flow in type II diabetic elderly patients when compared to healthy elderly.

## 2. Methods

### 2.1. Experimental Design

This was a controlled blind clinical trial, whose protocol was approved by the local Research Ethics Committee according to the Declaration of Helsinki, and all volunteers have signed the free and informed consent to participate in this study. The same researcher has performed the initial and final evaluation of measurements, who did not know to which group the participant belonged.

### 2.2. Subjects

Fourteen diabetic elderly women and fifteen healthy elderly women were allocated into diabetic or control groups. For including criteria, were selected women between 60 and 74 years, sedentary according the International Physical Activity Questionnaire (IPAQ-short form) [16]. For diabetic group, those who had medical diagnoses of type II diabetes for at least 2 complete years and who had used only oral hypoglycemic agents of biguanides class (metformin extended release, XR) in dosage between 500 and 850 mg were considered. For healthy elderly group, elderly women without self-reported diseases were considered.

Diabetic volunteers who have ingested the breakfast less than 2 hours or more than 3 hours and those who have taken metformin less than 2 hours before the intervention are excluded. For all volunteers, the complementary exclusion criteria were the current or past smoking or alcoholic habits, the cardiovascular, liver, or neuromuscular diseases, participants with prior labyrinthitis or embolic events, those who have had any surgery in last year, volunteers with metallic prosthesis as pacemaker, pins, or plates, advanced osteoporosis, or those who had difficulties during the evaluation or acute protocol.

### 2.3. Assessment of Measures

A blind researcher trained in test protocols has performed the initial glucose and lactate evaluations as primary outcomes, with the subject comfortably sitting in a room with a controlled temperature between 21 and 23°C. The volunteers have performed the hand cleaner with alcohol 70° Gay-Lussac before the digital puncture with disposable lancets. A blood drop was collected in the specific strip for the home glucose monitoring AccuChek Active® (Roche Diagnostics GmbH, Mannheim, Germany) according to International Organization for Standardization (ISO) recommendations in 2013 [17].

As secondary outcomes, all parameters were used to monitor the exercise intensity. The seric blood lactate with another blood drop was measured, analyzing in a portable lactometer, Accutrend Plus® (Roche Accutrend Plus, New York, USA), the heart rate (HR) that was collected by a digital oximeter Onyx (Nonim, model 9500, USA) and also the subject answer about the Borg's Rating of Perceived Exertion Scale (RPE) was observed. All data were collected for the same blind evaluator before and immediately after the acute intervention, and the glucose and lactate levels were assessed in the same finger of the same hand.

### 2.4. WBV Protocol and Positioning

**Figure 1:**
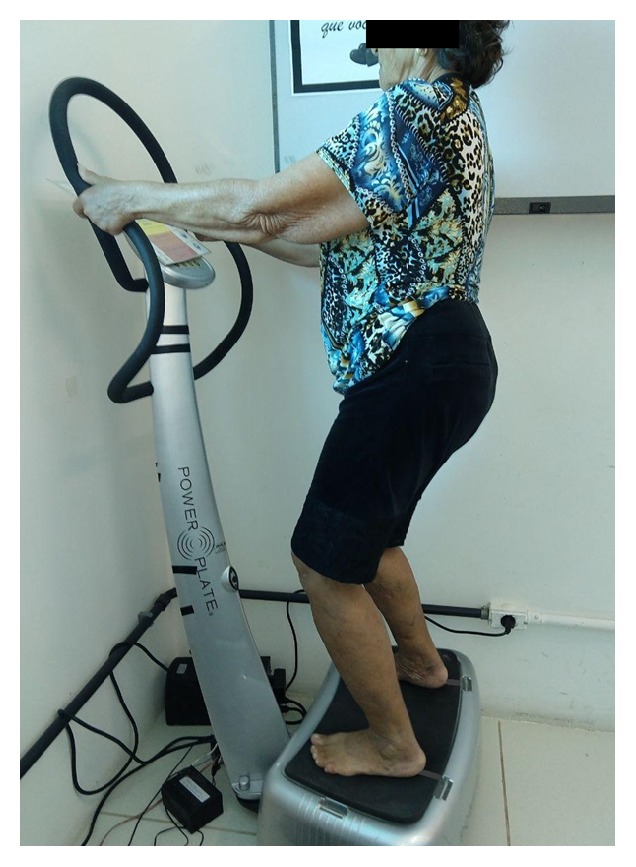
Positioning in vibrating platform for both groups.

After the first evaluation of parameters, all volunteers have performed a unique WBV session, while standing barefoot [18], with their heels off the ground, in static positioning of semisquats with 120° knee angle measured with a goniometer and arms extended, holding on platform, in order to minimize the axial transmission to the cranial base [19]. They were oriented to direct their heads and eyes forward and distributed their weight equally on both feet with a foot-to-foot distance of 20 centimeters, according to mark fixed on the plate platform (Figure 1).

The single session lasted 14 minutes and was composed of 10 bouts, lasting 2 minutes each one and the sets were separated by a 30-second rest period, with the participants remaining on the platform, in orthostatic position, with the feet at the same marked point.

The WBV was executed in a synchronic triaxial platform MY3 (Power Plate®, MY3, UK) that has a fixed frequency in 35 hertz and an amplitude in 4 millimeters and peak-to-peak displacement on 8 millimeters have been chosen, in order to generate the maximum load allowed by the platform. The peak acceleration was calculated in approximately 19.58 g [20].

### 2.5. Statistical Analysis

The sample size was determined by means of a sample calculation performed from the data collected during a pilot study with 5 volunteers, establishing a sample of 14 individuals for each group. The G-Power 3.1® statistical program was used (Behavior Research Methods, Instruments and Computers, Universitäd Kiel, Germany), considering a power (1 − *β*) of 95% and *α* = 5% to detect the difference between the groups and considering decreases around 20% on the glucose levels after intervention.

For the analysis of normality and homogeneity of the sample, the Kolmogorov-Smirnov and Levene tests were used. By the small sample, the Mann–Whitney test has been used for comparison between the groups and the Wilcoxon test for intragroup comparison was applied at the median and interquartile range. The analysis was conducted by the Statistical Package for the Social Sciences (SPSS) for Windows (version 20.0, Chicago, IL) and a significance level of *p* < 0.05 was established.

## 3. Results

**Figure 2:**
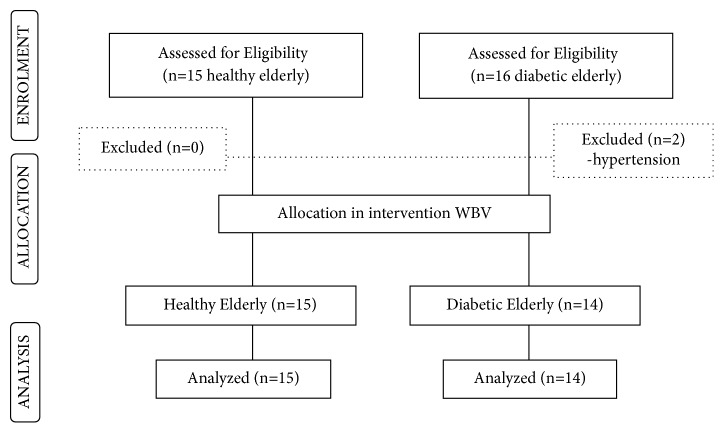
Flowchart of the study volunteers.

The study included 29 elderly women volunteers (15 controls and 14 diabetics), allocated in groups, according to Figure 2.

Before the training, the groups were homogeneous among themselves regarding the baseline characteristics, as shown in Table 1. However, the glucose pre values were higher in the diabetic group, as expected.

After the protocol, the intragroup comparison has shown differences in the HR, Borg, glucose, and lactate variables in the control (*p* = 0.009) and diabetic (*p* = 0.005) groups. The intergroup comparison has evaluated the post–post values between groups and was represented by *p* value in Table 2. The variation has shown the pre–post differences.

## 4. Discussion

The findings of the present study showed that the acute whole body vibration protocol could influence the blood glucose, when the protocol is applied in diabetic or not diabetic participants, with differences between them and this is the first investigation evaluating acute WBV protocol and the glycemic levels. The findings also show that after a WBV, as well as in any type of exercise, lactate, HR, and Borg values increased.

Ideally, interventions in diabetic patients should take a multiple approach and should include individual lifestyle-based prevention. Unfortunately, the number of individuals with TIIDM being increased so WHO established a global goal to implement healthy diets and to promote adequate levels of physical activity, aiming to reduce the risk or the complications in TIIDM [1].

According to the American Diabetes Association, it is recommended that older adults should do at least 150 minutes of moderate intensity or at least 75 minutes of vigorous-intensity aerobic physical activity throughout the week [21]. These ADA recommendations are important because, during physical exercise, several adaptive physiological responses occur, and the levels of the glucose uptake are influenced by the intensity and interval of physical exercise. The energy expenditure in activity starts with the glycogen and glucose consumption from liver and muscle [9], decreasing its values.

As an alternative to increase the physical activity in the elderly, the WBV is presented as whole body exercise at the same time with the advantage of joint protection [11] and has been shown as an effective option especially for diabetic patients. The results presented in this study indicate that an acute WBV protocol is efficient to reduce the glucose levels to the same value presented by healthy elderly, without differences between diabetic or not diabetic groups in post, making groups virtually similar, whereas it promotes a massive muscle contraction. Another beneficial effect of acute intervention is related to variation in glucose levels, with diabetic group showing larger decreases, with significant differences. The physiological response to the effect of WBV on glucose uptake of muscle cells is probably similar to the aerobic exercise that improves the GLUT4, making it easier for the glucose transport and increases the glycogen synthase and phosphorylase actions [22] in all muscles stimulated by vibrating platform. In this way, the results of present study corroborate from Del Pozo-Cruz et al. [23] data, which have shown advantageous effects on glycated hemoglobin, after 12 weeks of WBV training in diabetic patients. The maintained exercises, as have presented in both protocols, improve the glucose carriage to the muscle through the increase in muscle blood flow and opening in collateral capillary density [22]. Also, the acute WBV might auxiliary the glucose regulation by improving in beta cell function and in consequence the insulin resistance or enhance the sensitivity of muscle glucose uptake [24].

The newest proposal for explaining the changes in glycemic status after WBV is that the glucose levels could be modified through the interference in the skeletal system. The suggestion is that WBV supragravity impulse promotes increase in osteocalcin levels. Currently, the bone is considered as an endocrine organ, because the osteocalcin liberation can lead to lower blood glucose and increase in number of *β*-cell, insulin secretion, and insulin sensitivity [25]. Same clinical reports have confirmed that TIIDM patients have lower osteocalcin levels when compared to normal controls [25–27]. Moreover, exercise may stimulate increased secretion of osteocalcin by the bone secretion, and WBV more than other exercises could increase this response by its effect on anabolic bone responsiveness, since the supragravity stimuli activate the compressive loads in axial and appendicular bones, especially in lower limbs.

It is known that WBV could be an extenuating exercise for sedentary people, being the lactate levels an exercise intensity marker. This explains the modifications between the lactate pre and post values, since that the tonic-vibratory reflex in WBV, at first, sensitizes the afferent fibers Ia, with high oxidative capacity. After the recruitment of all these fibers, the *α*-motor neurons activate the muscular fibers type II, with low oxidative capacity and high glycolytic capacity, and these fibers are large lactate producers [19]. In this way, at the same time that the glucose is broken to offer energy for muscle work, decreasing their levels on blood, the lactate production is increasing and, for minimizing damage in muscle cells, is permeated to blood in a direction to the liver.

Also it is recognized that diabetics may be subject to premature muscle fatigue, even if without diabetic peripheral neuropathy or peripheral artery disease [7]. The mechanisms likely explaining the greater muscle fatigue in diabetic participants remain unclear and it has been suggested that this perception is related to changes in fiber metabolism and muscle composition [28], because sedentary habits could promote changes in type fibers. However, the findings of the present study could auxiliary to clarify this process, since that after a WBV protocol the lactate variation has presented differences. The diabetic group has shown the lowest levels of blood lactate, meaning that this substrate remained inside the muscle, and this could increase the fatigue since that the lactate originated by lactic acid is not injurious, but the H^+^ resulting from the breakdown of acid could be responsible for muscle suffer.

The same explanation connects the lactate levels and the Borg's RPE results. The subjective perception of effort is considered as a physiological indicator of physical stress and their values after the WBV provide indirect information about the muscle effort [29]. The subjective perception of effort takes into consideration that the H^+^ accumulation that follows the muscle lactacidemia causes muscle pain, according to Babraj and Hawkey in 2017 [30]. They have shown that a short-term of whole body vibration intervention improves the insulin sensitivity and raises the lactate, corroborating these findings, where the large lactate drainage to the blood in healthy elderly group means that less lactate remains into cells, decreasing the muscle hurt and RPE.

Some studies corroborate our findings in relation to the HR raise after WBV, and those results are age, sex, and activity independent. Kang et al. in 2016 [31] verified that 08 active young men and 08 active young women have shown increase in HR after eight weeks of WBV training. Licurci et al. in 2017 [32] verified that 07 old men and 04 old women also increased the HR after WBV sessions. Parra et al. in 2018 [33] found the same results, with 09 sedentary young women and 05 sedentary young men after Yoga movements in vibrating platform. The large muscle groups involving in WBV exercise at the same time that demands increases in HR in direct proportion to the effort intensity could explain these results.

The study limitations were mainly related to the control of physical activity and nutrition prior to each testing day. The use of accelerometers summed to provision of standard meals before the evaluation would improve the control over physical activity level and nutrition condition. Despite these limitations, it is believed that the results have not been compromised, whereas all participants were previously informed about the need not to perform strenuous exercises on the day of the evaluation, as well as not changing their nutritional routine.

It is important to observe that WBV exposure in healthy and diabetic elderly participants, after a relative short time of exposure, caused decrease in glucose level in both groups. This response supports a favorable response of WBV on glycemic control, besides the shortest intervention time, and it could be useful for population with reduced mobility. From a clinical point of view, it would be stimulating to observe in future studies the effects of WBV in elderly TIIDM whose use of drugs could be reduced.

## 5. Conclusion

Many elderly diabetic patients have sedentary lifestyle because of pain or joint injury, even knowing about the physical activity benefits. Our study revealed that WBV training in TIIDM has had significant beneficial effects on the control of glucose levels, still in an acute session. So that, the complete training probably will show better results about glycemic control and this finding could be especially important when prescribing exercise for elderly who are unable or unwilling to use traditional loads or who show poor exercise compliance. Furthermore, WBV requires significantly less time than conventional training and, therefore, reached a satisfactory compliance in previously inactive patients.

## Figures and Tables

**Table 1:** Characteristics of control and diabetic groups at baseline.

Variables	Control Group	Diabetes Group	*p* value
	(*n* = 15)	(*n* = 14)	
Age (years)	65.6 (63; 68.7)	66.6 (63; 68.7)	0.867
Weight (kg)	71.3 (60.2; 80.5)	70.1 (64.5; 72.2)	0.898
Height (cm)	162.6 (160.7; 165.5)	163 (158.2; 167.7)	0.858
BMI (kg/m^2^)	26.3 (22.5; 30.0)	25.4 (24.8; 27.0)	0.788
HR pre (bpm)	75 (69; 80)	71 (66.5; 76)	0.563
Borg exertion pre	0 (0; 0)	0 (0; 0)	1.00
Glucose pre (mg/dL)	97.5 (88.2; 105.2)	125.5 (109.5; 151.7)	**0.041** ^*∗*^
Lactate pre (mmol/L)	1.6 (0.9; 2.9)	1.3 (0.8; 2.0)	0.149

BMI: body mass index; HR: heart rate; kg: kilogram; cm: centimeters; m^2^: square meter; bpm: beats per minute; mg/dL: milligram per decilitre; mmol/L: millimole per litre. Data have been shown as median and interquartile range; ^*∗*^*p* < 0.05: control versusdiabetes.

**Table 2:** Comparison between intra- and intergroups after intervention.

Variables	Control Group	Diabetes Group	*p* value
	(*n* = 15)	(*n* = 14)	
HR pre (bpm)	75 (69; 80)	71 (66.5; 76)	0.563
HR post (bpm)	137 (119; 142)^††^	130 (123.5; 131.2)^††^	0.142
HR variation (bpm)	59 (41; 73.2)	59.5 (39.5; 66.2)	0.788
Borg exertion pre	0 (0; 0)	0 (0; 0)	1.00
Borg exertion post	4 (3; 5)^††^	4.5 (3; 6)^††^	**0.045** ^*∗*^
Borg exertion variation	4 (3; 5)	4.5 (3; 6)	**0.045** ^*∗*^
Glucose pre (mg/dL)	97.5 (88.2; 105.2)	125.5 (109.5; 151.7)	**0.041** ^*∗*^
Glucose post (mg/dL)	84.5 (78.7; 94.5)^††^	92.5 (87.7; 101.2)^††^	0.130
Glucose variation (mg/dL)	10.5 (6.5; 16.7)	26 (13.7; 45.2)	**0.010** ^*∗*^
Lactate pre (mmol/L)	1.6 (0.9; 2.9)	1.3 (0.8; 2.0)	0.149
Lactate post (mmol/L)	4.7 (4.3; 4.6)^††^	4.4 (4.3; 4.6)^††^	0.156
Lactate variation (mmol/L)	2.9 (2.5; 3.1)	2.4 (2.0; 2.9)	**0.033** ^*∗*^

HR: heart rate bpm: beats per minute; mg/dL: milligram per decilitre; mmol/L: millimole per litre. Data were presented as median and interquartile range; ^**∗**^*p* < 0.05: control versus diabetes; ^††^*p* < 0.05: pre versus post.
